# Identification of Regulatory Factors and Prognostic Markers in Amyotrophic Lateral Sclerosis

**DOI:** 10.3390/antiox11020303

**Published:** 2022-02-01

**Authors:** Hualin Sun, Ming Li, Yanan Ji, Jianwei Zhu, Zehao Chen, Lilei Zhang, Chunyan Deng, Qiong Cheng, Wei Wang, Yuntian Shen, Dingding Shen

**Affiliations:** 1 Key Laboratory of Neuroregeneration of Jiangsu and Ministry of Education, Co-Innovation Center of Neuroregeneration, NMPA Key Laboratory for Research and Evaluation of Tissue Engineering Technology Products, Jiangsu Clinical Medicine Center of Tissue Engineering and Nerve Injury Repair, Nantong University, Nantong 226001, China; sunhl@ntu.edu.cn (H.S.); 2022310030@stmail.ntu.edu.cn (Y.J.); 2022310042@stmail.ntu.edu.cn (Z.C.); 1924310007@stmail.ntu.edu.cn (L.Z.); 2122310008@stmail.ntu.edu.cn (C.D.); cq1981@ntu.edu.cn (Q.C.); ntwangwei911@ntu.edu.cn (W.W.); 2 Department of Laboratory Medicine, Binhai County People’s Hospital Affiliated to Kangda College of Nanjing Medical University, Yancheng 224500, China; liming861026@163.com; 3 Department of Orthopedics, Affiliated Hospital of Nantong University, Nantong 226001, China; zhujianwei@ntu.edu.cn; 4 Department of Pathology, Affiliated Hospital of Nantong University, Nantong 226001, China; 5 Department of Neurology, Shanghai Ruijin Hospital, School of Medicine, Shanghai Jiao Tong University, Shanghai 200025, China

**Keywords:** ALS, immune, inflammation, regulatory factors, prognostic markers

## Abstract

Amyotrophic lateral sclerosis (ALS) is a fatal neurodegenerative disease characterized by the progressive degeneration of motor neurons, leading to muscle atrophy, paralysis and even death. Immune disorder, redox imbalance, autophagy disorder, and iron homeostasis disorder have been shown to play critical roles in the pathogenesis of ALS. However, the exact pathogenic genes and the underlying mechanism of ALS remain unclear. The purpose of this study was to screen for pathogenic regulatory genes and prognostic markers in ALS using bioinformatics methods. We used Gene Ontology (GO) analysis, Kyoto Encyclopedia of Genes and Genomes (KEGG) analysis, gene set enrichment analysis (GSEA), and expression regulation network analysis to investigate the function of differentially expressed genes in the nerve tissue, lymphoid tissue, and whole blood of patients with ALS. Our results showed that the up-regulated genes were mainly involved in immune regulation and inflammation, and the down-regulated genes were mainly involved in energy metabolism and redox processes. Eleven up-regulated transcription factors (CEBPB, CEBPD, STAT5A, STAT6, RUNX1, REL, SMAD3, GABPB2, FOXO1, PAX6, and FOXJ1) and one down-regulated transcription factor (NOG) in the nerve tissue of patients with ALS likely play important regulatory roles in the pathogenesis of ALS. Based on construction and evaluation of the ALS biomarker screening model, cluster analysis of the identified characteristic genes, univariate Cox proportional hazards regression analysis, and the random survival forest algorithm, we found that MAEA, TPST1, IFNGR2, and ALAS2 may be prognostic markers regarding the survival of ALS patients. High expression of MAEA, TPST1, and IFNGR2 and low expression of ALAS2 in ALS patients may be closely related to short survival of ALS patients. Taken together, our results indicate that immune disorders, inflammation, energy metabolism, and redox imbalance may be the important pathogenic factors of ALS. CEBPB, CEBPD, STAT5A, STAT6, RUNX1, REL, SMAD3, GABPB2, FOXO1, PAX6, FOXJ1, and NOG may be important regulatory factors linked to the pathogenesis of ALS. MAEA, TPST1, IFNGR2, and ALAS2 are potential important ALS prognostic markers. Our findings provide evidence on the pathogenesis of ALS, potential targets for the development of new drugs for ALS, and important markers for predicting ALS prognosis.

## 1. Introduction

Amyotrophic lateral sclerosis (ALS) is a fatal neurodegenerative disease characterized by progressive muscle paralysis, which involves the degeneration of upper motor neurons (UMN) and lower motor neurons (LMN) in the motor cortex, brainstem, and spinal cord [1]. ALS is the most common adult motor neuron disease, with an incidence rate of 2 cases per 100,000 people and a prevalence of 5.4 cases per 100,000 people [2]. The mean age of onset of ALS is between 50 and 65 years. On average, death occurs because of respiratory failure within 2–3 years of onset [3]. There are two types of ALS: familial ALS (fALS, approximately 10% of cases) and sporadic ALS (sALS, approximately 90% of cases) [4]. Currently, there is no cure for ALS. Riluzole and edaravone are two drugs approved by the US Food and Drug Administration for ALS treatment. Riluzole is considered to reduce motor neuron damage by inhibiting glutamate release, while edaravone is considered to be a neuroprotective agent and free radical scavenger that reduces oxidative stress [5]. However, the molecular mechanism underlying the pathogenesis of ALS remains unclear, which limits the development of therapeutic methods. 

After years of research, many new discoveries have been made regarding the pathogenesis of ALS. Various environmental factors have been proposed to be associated with ALS, such as smoking, low antioxidant intake, intense physical exercise, head trauma, inflammation, poor nutritional status, and exposure to electromagnetic fields [6,7,8]. Immune imbalance greatly contributes to the pathogenesis of ALS, and activation and proliferation of many immune cells have been reported to increase with the progress of the disease [9]. The delicate balance between anti- and pro-inflammatory factors has been reported to regulate ALS progression and survival time, and turning the balance in favor of anti-inflammatory processes can help slow down the progression of this destructive disease [10]. Autophagy and mitochondrial autophagy, which are important mechanisms for maintaining neuronal homeostasis, are often impaired in ALS [11]. Autophagy is a stress response. Maintaining its balance may be the key to the pathogenesis and treatment of ALS. ALS patients often have mutations in genes involved in autophagy that affect various processes of autophagy, including autophagy initiation, autophagosome maturation and transport, endosome transport, and autophagosome–lysosome fusion [12]. Evidence indicates that oxidative stress may be increased in sALS and fALS patients, which may affect mitochondrial function, aggravate endoplasmic reticulum stress, and affect protein homeostasis, eventually leading to cell damage and neuron loss [13]. Mitochondrial dysfunction is a core component of ALS. ALS-related mitochondrial dysfunction has many manifestations, including oxidative phosphorylation deficiency, generation of reactive oxygen species, impaired calcium buffering capacity, and defective mitochondrial dynamics [14]. Protein aggregation in ALS also affects the pathogenesis in terms of self-aggregation of prion-like domains, altered RNA molecule formation, and dysfunction of the protein quality control system [15]. The fact that ALS is a syndrome involving multiple causes is a key reason its exact pathogenesis has not yet been clarified.

Abnormal expression of many genes has been shown to play an important role in the occurrence and development of ALS. Superoxide dismutase 1 (SOD1) is a small protein that contributes to partial resistance to oxidative stress [16]. Approximately 15% of fALS cases are caused by *SOD1* mutations [17]. Additionally, 40–50% of fALS cases and 10% of sALS cases are caused by nucleotide repeat expansion in the *C9orf72* gene [17]. TAR DNA-binding protein 43 (TDP-43) is a multifunctional DNA/RNA-binding protein involved in RNA-related metabolism [18]. The hallmark pathological feature of most ALS cases is the presence of abnormal ubiquitinated proteins, especially TDP-43, in neuronal cytoplasmic inclusions [19]. Mutation of cyclin F (encoded by CCNF) can cause abnormal ubiquitination and the accumulation of ubiquitinated proteins, including TDP-43 (18). Furthermore, TANK-binding kinase 1 (TBK1) is an important involved in the innate immune response. An exome sequencing study found that TBK1 was greatly correlated with ALS, and TBK1 may play a key role in autophagosome maturation and the clearance of pathological aggregates [20]. A genome-wide association study identified a new risk site on chromosome 21 and ascertained that the *C21orf2* gene increases the risk of developing ALS [21]. Matrin-3 (MATR3), which is a 125-kDa nuclear matrix protein that interacts with TDP-43 [22], has been observed in the nucleus, and occasionally the cytoplasm, of motor neurons in patients with ALS. Additionally, MATR3-positive cytoplasmic inclusions, which are rare in the general population, have been detected in ALS patients with repeat expansion in *C9orf72* [23]. As ALS is a syndrome involving multigene expression dysregulation, in this study, we screened for possible pathogenic genes involved in ALS using bioinformatics methods. 

In this study, we used Gene Ontology (GO) analysis, Kyoto Encyclopedia of Genes and Genomes (KEGG) analysis, gene set enrichment analysis (GSEA), and expression regulation network analysis to investigate the function of differentially expressed genes (DEGs) in the nerve tissue, lymphoid tissue, and whole blood of patients with ALS. We identified 12 transcription factors that play important regulatory roles in the pathogenesis of ALS and four prognostic genes that affect the survival of ALS patients. This is highly significant for the development of targeted treatment and prognostic tools for ALS in the future. Thus, our study linked DEGs in ALS, GO, KEGG, and GSEA analysis, and an expression regulation network, which will help to develop new targets for the treatment of ALS. 

## 2. Materials and Methods

### 2.1. Sources of mRNA Microarray and Sequencing Data from Patients with ALS

Data were sourced from Gene Expression Omnibus (GEO). The data were RNA expression data obtained from samples harvested from nerve tissue (motor neurons of the lumbar spinal cord, frontal cortex, cerebellum, and motor cortex in snap-frozen brain blocks), lymphoid tissue, and whole blood. Detection platforms and sample sizes are shown in Supplementary Table S1. 

### 2.2. Bioinformatics Analysis

Using Pearson’s correlation coefficient, we assessed the correlations of DEGs among the datasets (Supplementary Table S1). As the data were collected by different platforms (chips and RNA sequencing) and sample sizes were inconsistent, the correlations between datasets could not be calculated directly based on expression levels. Instead, the correlations were calculated based on differential gene expression (log2 transformed fold change) and displayed in a heatmap.

We screened for DEGs in each dataset. The chip data were processed using the student t-test and modest t-test by the limma R package, and the union of the result obtained by both methods was selected. The sequencing data were processed using edgeR and DEseq2 R package, and the union of the data processed by both methods were selected. The thresholds for DEGs were as follows: fold change ≥1.3 and *p* < 0.05 for nerve tissue, and fold change ≥1.2 and *p* < 0.05 for lymphoid tissue and whole blood. 

### 2.3. Functional Enrichment Analysis of DEGs 

First, the DEGs in each dataset were divided into up-regulated genes and down-regulated genes, which were enriched and analyzed respectively, and the enriched KEGG pathways with *p* < 0.05 in each dataset were considered to be significantly enriched. Second, the DEGs (defined as sel2g) that were regarded as differential expressed in at least two datasets were identified for subsequent analysis. These DEGS were consistently up-regulated (sel2g.up) or consistently down-regulated (sel2g.down). Thereafter, these common DEGs were subjected to GO analysis (in terms of biological processes [BPs], molecular functions [MFs], and cellular components [CCs]) and KEGG analysis, using the sel2g data. In addition, functional enrichment analysis, comprising GO BP and KEGG analysis, of the intersection of sel2g and DEGs in lymph or whole blood tissues were performed.

### 2.4. Expression Regulation Network Analysis

Expression regulation network analysis was performed to screen for important regulatory factors (i.e., transcription factors) among the DEGs in the nerve tissue of patients with ALS, and to analyze the gene expression regulation relationships between genes. Gene regulation relationship data, i.e., “controls-expression-of”, were sourced from the Pathway Commons database (http://www.pathwaycommons.org/pc/home.do, June 15, 2021). Subnetworks were identified from the networks of up- and down-regulated genes using the Markov cluster algorithm (MCL) clustering function [24].

### 2.5. Preprocessing of Data for ALS Biomarker Screening

The data used for ALS biomarker screening were sourced from GEO (accession no. GSE112681). The data were from 397 ALS samples, 645 control samples. All these samples were from the blood. The chip platforms were Illumina HumanHT-12 v3.0 and v4.0. As the data were sourced from two chip platforms, only the same probes were retained. The outliers were removed by principal component analysis (PCA) and partial least square discriminant analysis (PLS-DA), and the batch effect were removed using the SVA R package. The genes that met the criteria for differential expression in ALS compared to control samples (*p* < 0.05) were selected for subsequent analysis. The differential expressed probsets found in both training and validation datasets were kept, and thus 4770 probes were retained.

### 2.6. Construction and Screening of ALS Biomarker Models

After eliminating 35 samples (due to outliers), there were 759 and 248 samples in the training and validation datasets, respectively. The training dataset was used for cross-validation by dividing the samples into two groups: one for training and the other for model evaluation. More precisely, using 5-fold cross-validation, the samples were split into five groups, and four were used for training while the remaining one was used for validation. As the procedure was performed five times, each group had the opportunity to become a training dataset to evaluate the performance of the classifiers. The mean model accuracy was determined across the five groups. The genes associated with high model accuracy were selected as the characteristic genes. The second classifiers were AdaBoost, Random Forest, and support vector machines (SVM). These classifiers were constructed with the identified characteristic genes and their classification accuracy in the training dataset was evaluated. The classification accuracy of the SVM classifier was highest. However, whether there was a difference in both groups (ALS vs. Control) was not considered when initially identifying the characteristic genes. Therefore, the SigFeature R package was used to further screen for characteristic genes with differences in both groups (ALS vs. Control) and strong classification performance in the second classifiers. Finally, the constructed ALS biomarker models were evaluated based on accuracy, precision, recall, and receiver operating characteristic (ROC) curve analysis.

### 2.7. Survival Analysis of ALS Biomarkers

The data used for survival analysis were from 290 patients in the training dataset and 94 patients in the validation dataset. First, we used univariate Cox proportional hazards regression to screen for survival-related markers (*p* < 0.05). Next, the random survival forest algorithm was used to further screen for markers with the highest correlation with survival (relative importance >0.5). Four key survival-related markers were obtained and then used to construct a multivariate Cox regression model. The regression coefficients of each marker were obtained. The following risk score formula was constructed to determine whether each patient was in the high- or low-risk group: 0.13 × ALAS2 + 0.17 × TPST1 + 0.17 × MAEA + 0.35 × IFNGR2. The risk score for each patient in the training dataset was calculated. The patients were classified according to the median risk score in the dataset. Patients with a risk score lower than the median were included in the low-risk group, and those with a risk score higher than the median were included in the high-risk group. Survival curves were drawn to compare the survival status between the low- and high-risk groups. In addition, a cluster heatmap and boxplots were used to show the expression of the four survival-related genes in the low- and high-risk groups.

### 2.8. Statistical Analysis 

Univariate and multivariate Cox regression analysis were used to analyze the survival of ALS patients based on potential prognostic factors such as age, sex, site, and risk score. GO and KEGG analysis were performed to calculate enrichment *p*-values using hypergeometric distribution tests.

## 3. Results

### 3.1. Functional Annotation of DEGs in Nerve Tissue of Patients with ALS

**Figure 1:**
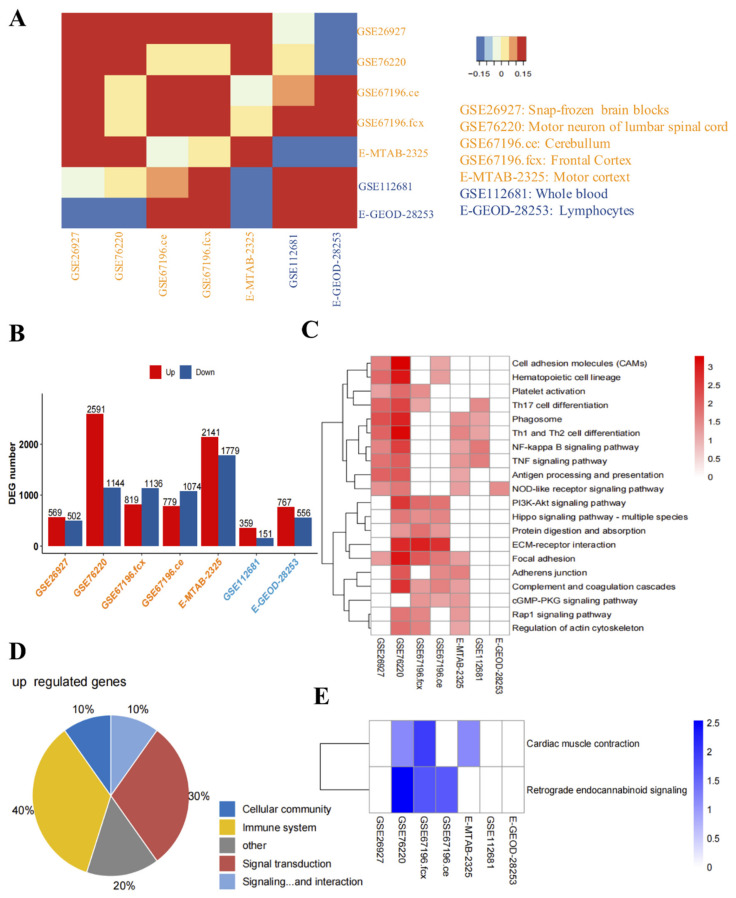
Identification and functional analysis of the differentially expressed genes (DEGs) in the nerve tissue, lymphoid tissue, and whole blood of amyotrophic lateral sclerosis (ALS) patients. (**A**) Correlation analysis of the DEGs in the nerve tissue, lymphoid tissue, and whole blood of ALS patients. The color from blue to yellow to red represents the change of the correlation coefficient from negative to positive correlation. (**B**) Number of DEGs in the nerve tissue, lymphoid tissue, and whole blood of ALS patients. The horizontal axis represents the GEO number. (**C**) Enriched Kyoto Encyclopedia of Genes and Genomes (KEGG) pathways of the genes up-regulated in the nerve tissue, lymphoid tissue, and whole blood. The color from white to red represents the gradual change of *p*-value from high to low. If *p* < 0.05 is not satisfied, it is indicated in white. (**D**) Pie chart of the proportion of the genes up-regulated in various KEGG pathways in the nerve tissue, lymphoid tissue, and whole blood of ALS patients. (**E**) Enriched KEGG pathways of the genes down-regulated in the nerve tissue, lymphoid tissue, and whole blood. The color from white to blue represents the gradual change of *p*-value from high to low. If *p* < 0.05 is not satisfied, it is indicated in white.

To better understand the relationships of the DEGs among datasets, we analyzed the correlations between them. We found that the similarity of DEGs between datasets on nerve tissue (Figure 1A) and the number of DEGs in nerve tissue in each dataset were higher than the corresponding values for lymphoid tissue and whole blood (Figure 1B). 

To investigate the relationship between the DEGs and the occurrence and development of ALS, we performed KEGG analysis and found that the up-regulated genes in the nerve tissue of ALS patients were mainly related to immunity and inflammation (such as cell adhesion molecules, hematopoietic cell lineage, Th17 cell differentiation, phagosome, Th1 and Th2 cell differentiation, NF-κB signaling pathway, TNF signaling pathway, NOD-like receptor signaling pathway, and antigen processing and presentation). In addition, the up-regulated genes in whole blood were mainly related to phagosome, Th1 and Th2 cell differentiation, NF-κB signaling pathway, and TNF signaling pathway, while those in lymphoid tissue were mainly related to the NOD-like receptor signaling pathway (Figure 1C). Figure 1D shows that the pathways in which the DEGs were involved were mainly related to immune system and signal transduction. The pathways in which down-regulated genes were involved were mainly related to cardiac muscle contraction and retrograde endocannabinoid signaling (Figure 1E). 

**Figure 2:**
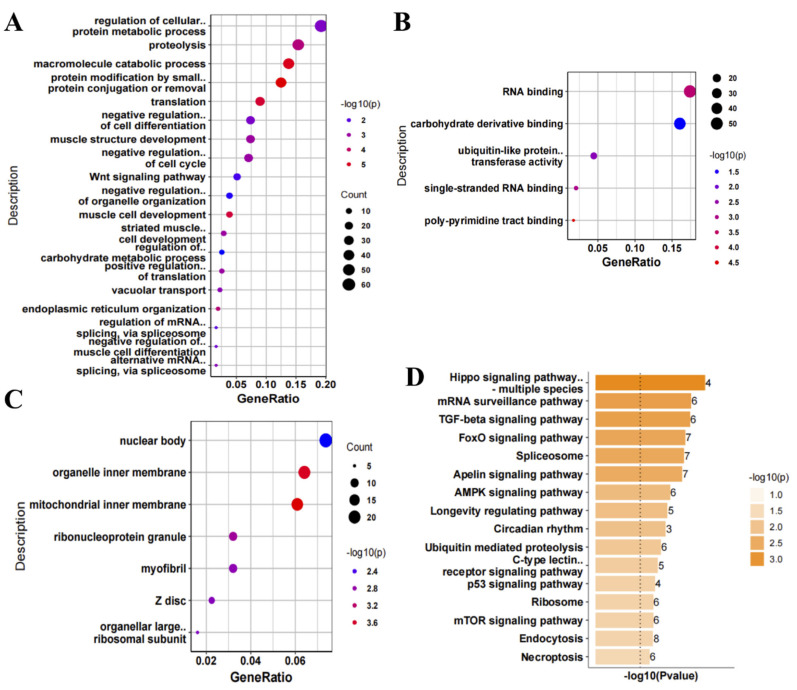
Functional enrichment analysis of up-regulated genes in nerve tissue of amyotrophic lateral sclerosis (ALS) patients. (**A**) Gene Ontology (GO) biological process, (**B**) GO molecular function, (**C**) GO cellular component, and (**D**) Kyoto Encyclopedia of Genes and Genomes (KEGG) analysis of up-regulated genes in the nerve tissue of ALS patients. The number on the right represents the number of differentially expressed genes.

**Figure 3:**
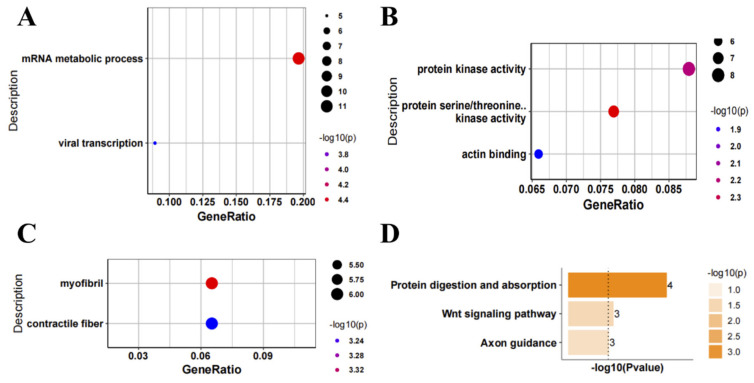
Functional enrichment analysis of down-regulated genes in nerve tissue of amyotrophic lateral sclerosis (ALS) patients. (**A**) Gene Ontology (GO) biological process, (**B**) GO molecular function, (**C**) GO cellular component, and (**D**) Kyoto Encyclopedia of Genes and Genomes (KEGG) analysis of down-regulated genes in nerve tissue of patients with ALS. The number on the right represents the number of differentially expressed genes.

As our results showed that most DEGs were in the nerve tissue of ALS patients, we performed GO and KEGG analysis of the up- and down-regulated genes in the nerve tissue of ALS patients. Regarding the GO BPs, the up-regulated genes were mainly related to proteolysis, macromolecule catabolic process, protein modification and translation, negative regulation of cell differentiation, and negative regulation of cell cycle (Figure 2A). The GO MFs mainly included RNA binding (Figure 2B), and the GO CCs mainly included organelle inner membrane and mitochondrial inner membrane (Figure 2C). Regarding the KEGG pathways, the up-regulated genes were mainly involved in the Hippo signaling pathway, mRNA surveillance pathway, transforming growth factor (TGF)-β signaling pathway, FoxO signaling pathway, and spliceosome (Figure 2D). Regarding the GO BPs, the down-regulated genes were mainly involved in the mRNA metabolic process (Figure 3A). The GO MFs mainly included protein kinase activity, protein serine/threonine kinase activity, and actin binding (Figure 3B), while the GO CCs mainly included myofibril and contractual fiber (Figure 3C). Regarding the KEGG pathways, the down-regulated genes were mainly involved in protein digestion and absorption and the Wnt signaling pathway (Figure 3D).

**Figure 4:**
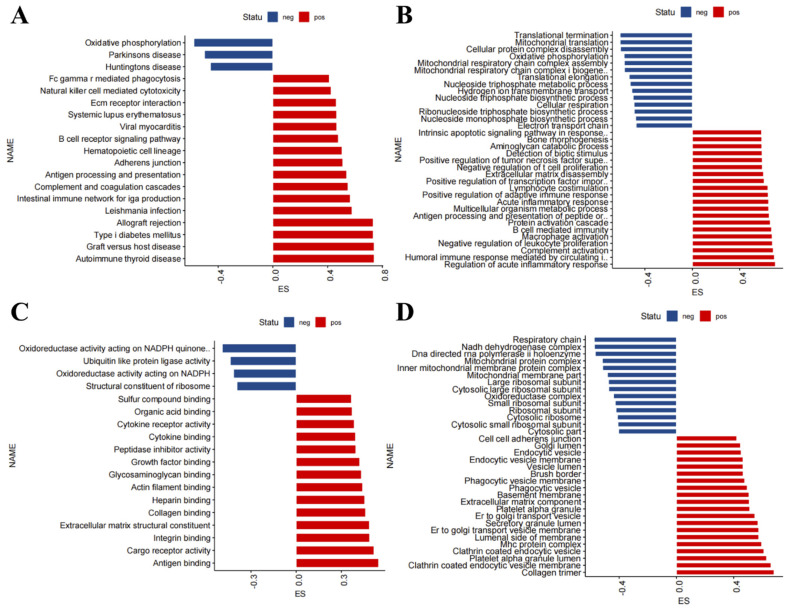
Gene set enrichment analysis (GSEA) of differentially expressed genes (DEGs) in the nerve tissue of amyotrophic lateral sclerosis (ALS) patients. GSEA of DEGs in the nerve tissue of ALS patients based on (**A**) KEGG pathways, (**B**) Gene Ontology (GO) biological process terms, (**C**) GO molecular function terms, and (**D**) GO cellular component terms.

We performed GSEA to analyze the function of the DEGs in the nerve tissue of ALS patients. KEGG analysis revealed that GSEA positive was mainly related to immunity, inflammation, and cell phagocytosis (for example, autoimmune thyroid disease, graft versus host disease, allograft rejection, internal immune network for IgA production, antigen processing and presentation, B cell receiver signaling pathway, Fcγ receptor-mediated phagocytosis, and natural killer cell-mediated cytotoxicity), and GSEA negative was mainly related to oxidative phosphorylation and neurodegenerative diseases (such as Parkinson’s disease and Huntington’s disease) (Figure 4A). GO BP analysis revealed that GSEA positive was mainly related to immunity and inflammatory response (for example, regulation of acute inflammatory response, complement activation, macrophage activation, B cell-mediated immunity, acute inflammatory response, and positive regulation of adaptive immune response), and GSEA negative was mainly related to energy metabolism (for example, mitochondrial translation, oxidative phosphorylation, mitochondrial respiratory chain complex assembly, mitochondrial respiratory chain complex I biogenesis, and cellular respiration) (Figure 4B). GO MF analysis revealed that GSEA positive was mainly related to antigen binding, cargo receptor activity, integrity binding, cytokine binding, and cytokine receiver activity, and GSEA negative was mainly related to oxidoreductase activity acting on NADPH quinone, oxidoreductase activity acting on NADPH, and ubiquitin-like protein ligase activity (Figure 4C). GO CC analysis revealed that GSEA positive was mainly related to collagen trimer, clathrin-coated endocytic vesicle membrane, MHC protein complex, and phagocytic vesicle, and GSEA negative was mainly related to respiratory chain, NADH dehydrogenase complex, and mitochondrial protein complex (Figure 4D). Taking the results together, the conclusions we reached through various methods were basically consistent, i.e., the functions of the up-regulated genes in the nerve tissue of ALS patients were mainly related to immunity and inflammation, and the functions of the corresponding down-regulated genes were mainly related to energy metabolism. 

### 3.2. Functional Analysis of the DEGs Co-Existing in the Nerve Tissue, Lymphoid Tissue and Whole Blood

**Figure 5:**
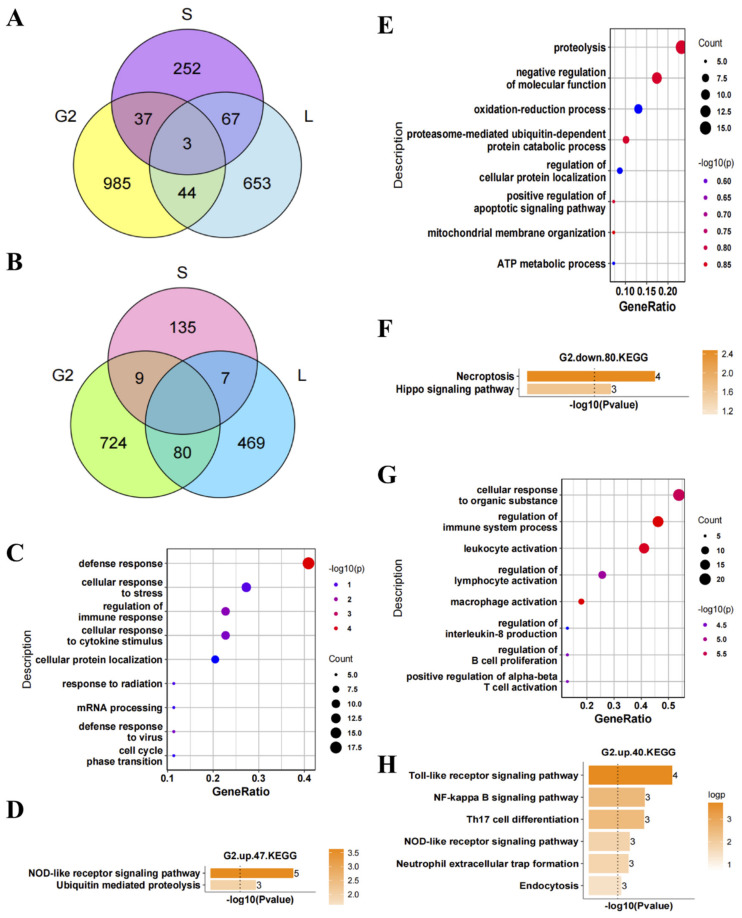
Identification of differentially expressed genes (DEGs) co-existing in the nerve tissue, lymphoid tissue, and whole blood of amyotrophic lateral sclerosis (ALS) patients and their functional enrichment analysis. (**A**) Venn diagram of the up-regulated genes in the nerve tissue, lymphoid tissue, and whole blood (G2: DEGs in nerve tissue of ALS patients (sel.2g), L: DEGs in lymphoid tissue of ALS patients, S: DEGs in whole blood of ALS patients). (**B**) Venn diagram of the down-regulated genes in the nerve tissue, lymphoid tissue, and whole blood of ALS patients. Gene Ontology (GO) biological process analysis and Kyoto Encyclopedia of Genes and Genomes (KEGG) analysis of the (**C**,**D**) up- and (**E**,**F**) down-regulated genes co-existing in the nerve tissue and lymphoid tissue of ALS patients and the (**G**,**H**) up-regulated genes co-existing in the nerve tissue and whole blood of ALS patients.

We analyzed the DEGs co-existing in the nerve tissue, lymphoid tissue, and whole blood and found that there were few of them (Figure 5A,B). First, we analyzed the function of the 47 up-regulated genes co-existing in nerve and lymphoid tissues. GO BP analysis revealed that these genes were mainly involved in the defense response, cellular response to stress, and regulation of immune response (Figure 5C). KEGG analysis showed that these genes were mainly involved in the NOD-like receptor signaling pathway and ubiquitin-mediated proteolysis pathway (Figure 5D). Second, we analyzed the function of the 80 down-regulated genes co-existing in the nerve and lymphoid tissues. GO BP analysis revealed that these genes were mainly involved in proteolysis, negative regulation of molecular function, and oxidation–reduction process (Figure 5E). KEGG analysis revealed that these genes were mainly involved in necroptosis and the Hippo signaling pathway (Figure 5F). Finally, we analyzed the function of the 40 up-regulated genes co-existing in the nerve tissue and whole blood. GO BP analysis revealed that these genes were mainly involved in the cellular response to organic substance, regulation of immune system process, regulation of lymphocyte activation, macrophage activation, interleukin (IL)-8 production, and leukocyte activation (Figure 5G). KEGG analysis revealed that these genes were mainly involved in the Toll-like receptor signaling pathway, NF-κB signaling pathway, and Th17 cell differentiation pathway (Figure 5H). There were very few down-regulated genes co-existing in the nerve tissue and whole blood, so functional analysis was not performed. Taking the results together, the up-regulated genes co-existing in the nerve tissue, lymphoid tissue, and whole blood were mainly involved in immunity and inflammation. The corresponding down-regulated genes were mainly involved in oxidation–reduction process and proteolysis. It can be inferred that the DEGs co-existing in the nerve tissue, lymphoid tissue, and whole blood may be the important regulatory factors for the occurrence and development of ALS. 

### 3.3. Important Regulatory Factors in ALS

**Figure 6:**
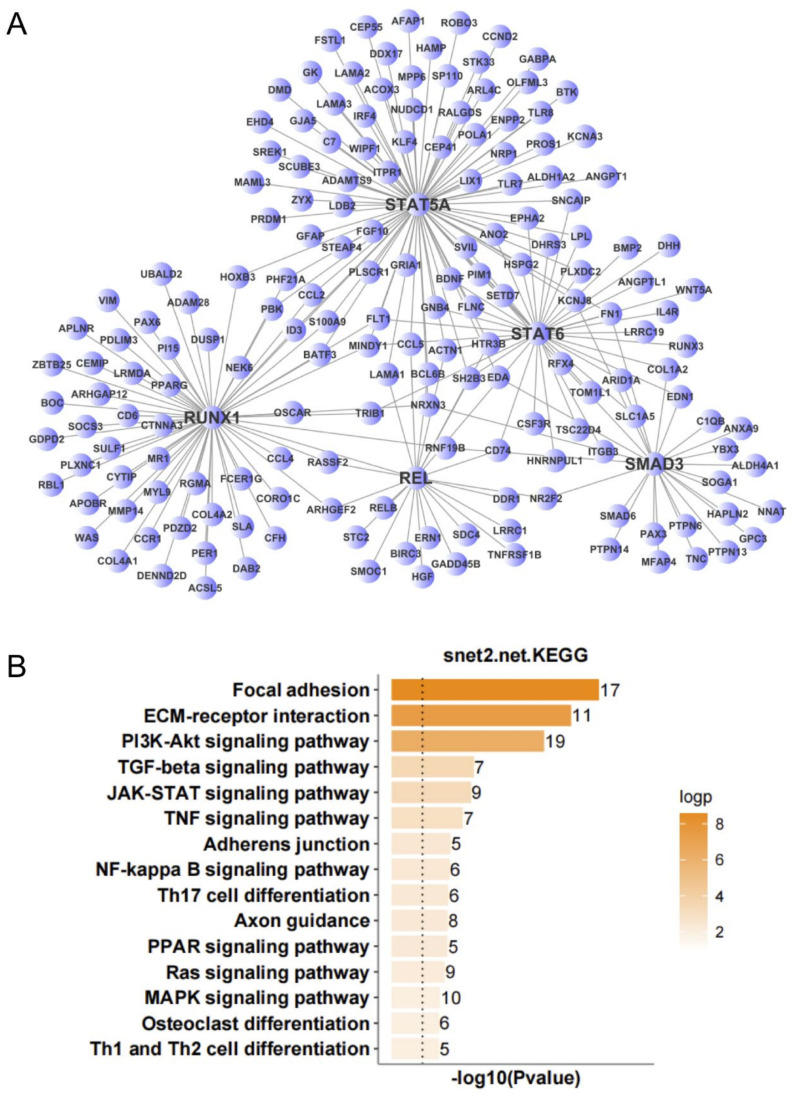
Expression regulation network and Kyoto Encyclopedia of Genes and Genomes (KEGG) analysis of up-regulated genes in nerve tissue of amyotrophic lateral sclerosis (ALS) patients. (**A**) Subnetwork with STAT5A, STAT6, RUNX1, REL, and SMAD3 as the main regulatory factors. (**B**) KEGG analysis of subnetwork target genes with STAT5A, STAT6, RUNX1, REL, and SMAD3 as the main regulatory factors.

**Figure 7:**
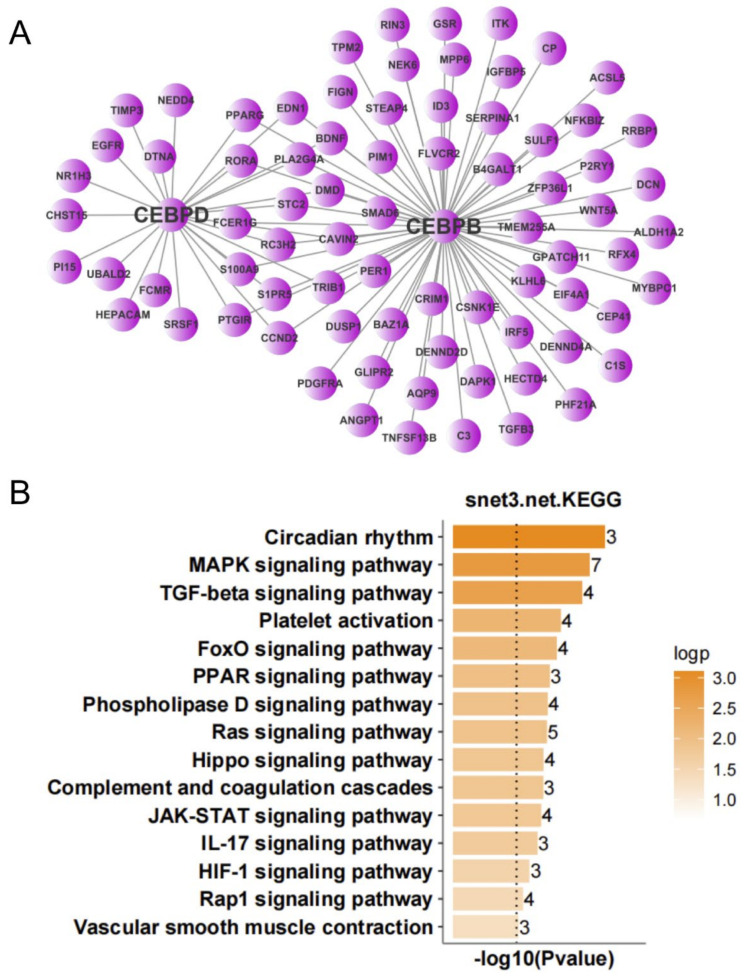
Expression regulation network and Kyoto Encyclopedia of Genes and Genomes (KEGG) analysis of up-regulated genes in nerve tissue of amyotrophic lateral sclerosis (ALS) patients. (**A**) Subnetwork with CEBPB and CEBPD as the main regulatory factors. (**B**) KEGG analysis of subnetwork target genes with CEBPB and CEBPD as the main regulatory factors.

**Figure 8:**
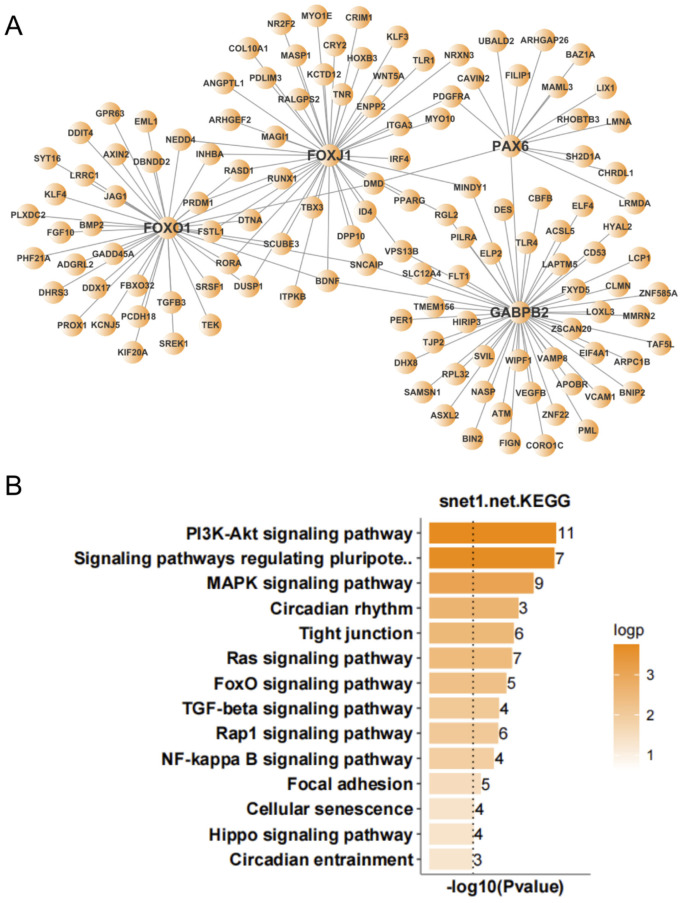
Expression regulation network and Kyoto Encyclopedia of Genes and Genomes (KEGG) analysis of up-regulated genes in nerve tissue of amyotrophic lateral sclerosis (ALS) patients. (**A**) Subnetwork with PAX6, FOXJ1, GABPB2, and FOXO1 as the main regulatory factors. (**B**) KEGG analysis of subnetwork target genes with PAX6, FOXJ1, GABPB2, and FOXO1 as the main regulatory factors.

**Figure 9:**
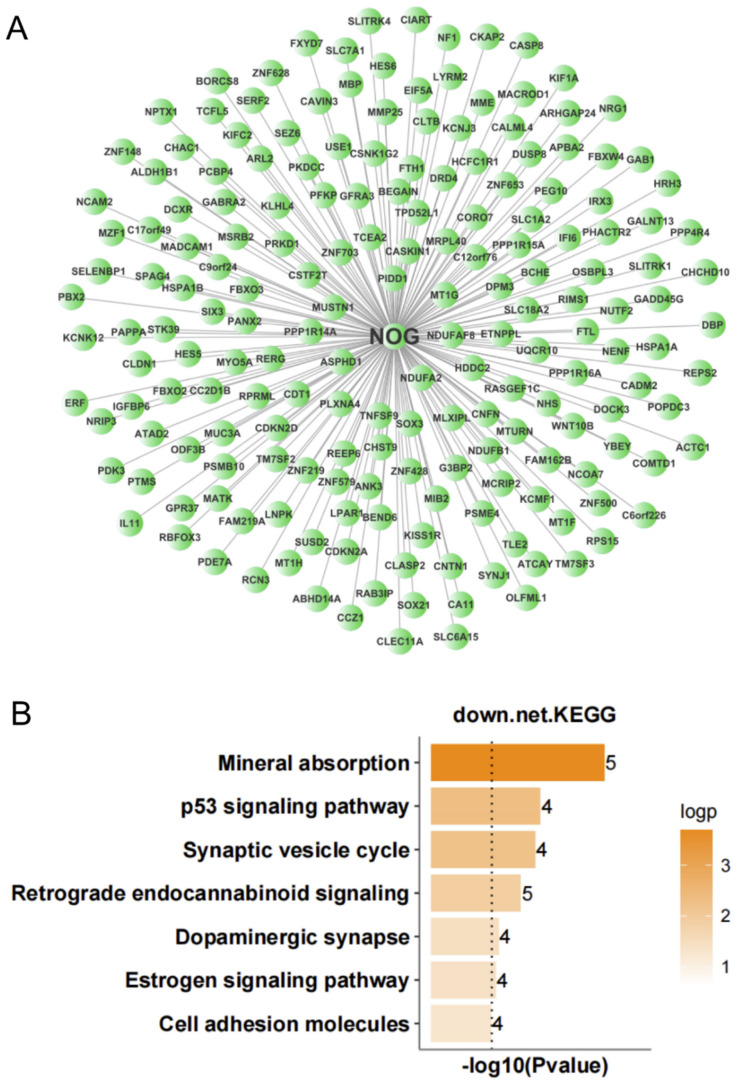
Expression regulation network and Kyoto Encyclopedia of Genes and Genomes (KEGG) analysis of down-regulated genes in nerve tissue of amyotrophic lateral sclerosis patients. (**A**) Network with NOG as the main regulatory factor. (**B**) KEGG analysis of network target genes with NOG as the main regulatory factor.

To further screen for transcription factors that may play important regulatory roles in the occurrence and development of ALS, we analyzed the expression regulatory relationships among the DEGs in the nerve tissue and screened for important transcription factors. According to the number of target genes of the transcription factors and the multiple of differential gene expression, we identified 12 important transcription factors (Table 1). An expression regulatory network of up- and down-regulated transcription factors was constructed by MCL clustering. The overlap among the target genes of these transcription factors was determined, and the transcription factors with high overlap were clustered into subnetworks. This resulted in the up-regulated transcription factors being clustered into three subnetworks. KEGG analysis was performed to analyze the function of target genes corresponding to each subnetwork. The target genes of subnetwork 1 (with STAT5A, STAT6, RUNX1, REL, and SMAD3 as the main regulatory factors) were mainly involved in focal adhesion, ECM-receptor interaction, PI3K-Akt signaling pathway, TGF-β signaling pathway, JAK-STAT signaling pathway, and TNF signaling pathway (Figure 6). The target genes of subnetwork 2 (with CEBPB and CEBPD as the main regulatory factors) were mainly involved in circadian rhythm, MAPK signaling pathway, TGF-β signaling pathway, platelet activation, FoxO signaling pathway, and PPAR signaling pathway (Figure 7). The target genes of subnetwork 3 (with PAX6, FOXJ1, GABPB2, and FOXO1 as the main regulatory factors) were mainly involved in the PI3K-Akt signaling pathway, signaling pathways regulating pluripotency of stem cells, MAPK signaling pathway, circadian rhythm, tight junction, Ras signaling pathway, and FoxO signaling pathway (Figure 8). Taking the results together, the 11 up-regulated transcription factors were mainly involved in the regulation of immunity and inflammation, oxidative stress, autophagy, and other related pathways. NOG, the single down-regulated transcription factor, mainly participated in mineral absorption, p53 signaling pathway, synaptic vesicle cycle, retrograde endocannabinoid signaling, and dopaminergic synapse (Figure 9), which are mainly related to the structure and function of synapses in the nervous system. 

### 3.4. ALS Biomarker Analysis

**Figure 10:**
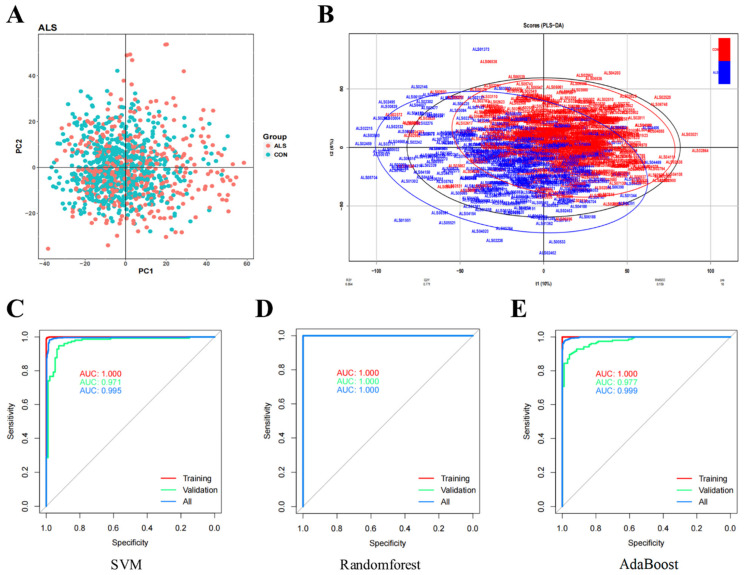
Screening for prognostic markers in amyotrophic lateral sclerosis and model evaluation. (**A**) Principal component analysis. (**B**) Partial least squares discriminant analysis. (**C**–**E**) Receiver operating characteristic (ROC) curves of three classifiers. The three lines represent three datasets (Training, Validation, All), respectively. Area under the curve (AUC) value reflects the ability of the classifier to correctly sort the samples.

PCA and PLS-DA were performed. PCA revealed that it was difficult to separate the samples between the ALS and control groups (Figure 10A). PLS-DA showed that the two groups of samples could be separated. A total of 35 ALS samples were eliminated (Figure 10B), because the 35 ALS samples mixed in the control subjects. Subsequently, we constructed ALS biomarker screening models and finally obtained 79 characteristic genes (Supplementary Table S2). Next, based on the 79 characteristic genes, we used three kinds of classifiers (SVM, Random Forest, and AdaBoost) to construct binary classification models. Their prediction results regarding sample classification were evaluated. All three classifiers led to good results in three datasets (Training, Validation, All) (Supplementary Table S3). The ROC curve analysis showed that the samples could be well separated using the characteristic genes (Figure 10C–E). 

Heatmap analysis of the training dataset showed that 50 characteristic genes were highly expressed in most ALS patients, while 29 were weakly expressed in most ALS patients (Supplementary Figure S1). Heatmap analysis of the validation dataset showed that 54 characteristic genes were highly expressed in most ALS patients, while 25 were weakly expressed in most ALS patients (Supplementary Figure S2). The heatmaps of the 79 characteristic genes were basically the same in the training and validation datasets: 49 characteristic genes were highly expressed in most ALS patients in both datasets, while 24 were weakly expressed in most ALS patients in both datasets.

**Figure 11:**
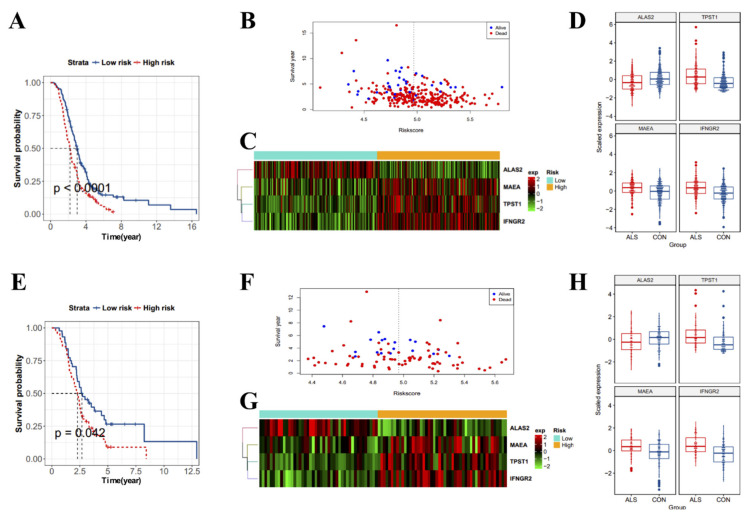
Analysis of prognostic markers in amyotrophic lateral sclerosis (ALS) patients. (**A**) Survival curves of ALS patients in the training dataset. (**B**) Analysis of ALS patients with different survival statuses and survival periods in the training dataset according to risk score. Red indicates the dead ALS patients and blue indicates the surviving ALS patients. The vertical line indicates the median risk score; the left side of the median is low risk, and the right side is high risk. (**C**) Heatmap of the expression of four survival-related genes (MAEA, TPST1, IFNGR2, and ALAS2) in ALS patients in the training dataset. Different colors indicate gene expression levels in different samples. (**D**) Boxplots of the expression of four survival-related genes in ALS patients in the training dataset. (**E**) Survival curves of ALS patients in the validation dataset. (**F**) Analysis of ALS patients with different survival statuses and survival periods according to risk score. Red indicates the dead ALS patients and blue indicates the surviving ALS patients. The vertical line indicates the median risk score; the left side of the median is low risk, and the right side is high risk. (**G**) Heatmap of the expression of four survival-related genes in ALS patients in the validation dataset. Different colors indicate gene expression levels in different samples. (**H**) Boxplots of the expression of four survival-related genes in ALS patients in the validation dataset.

To investigate the value of the 79 characteristic genes for predicting the prognosis of ALS patients, we used univariate Cox proportional hazards regression analysis to screen for markers related to the survival of ALS patients, and 18 survival-related markers were identified (*p* < 0.05) (Supplementary Table S4). Next, we used the random survival forest algorithm to screen for markers closely related to survival. Higher absolute values indicate a closer relationship with survival. Four key survival-related markers (MAEA, TPST1, IFNGR2, and ALAS2; relative importance >0.5) were obtained (Supplementary Table S5). Next, we performed survival analysis among the training and validation datasets. A risk score formula was constructed. According to the median risk score, patients were divided into low- and high-risk groups. Survival curves were constructed for the training dataset. In the training dataset, the survival of high-risk ALS patients was shorter than that of low-risk ALS patients (Figure 11A). We also visualized the distribution of survival status and survival period among patients with different risk scores, which demonstrated that surviving ALS patients tended to have a low risk score (Figure 11B). Next, we drew heatmaps and boxplots for the four risk survival-related markers to analyze their expression levels and found that MAEA, TPST1, and IFNGR2 were highly expressed in high-risk ALS patients, while ALAS2 was highly expressed in low-risk ALS patients (Figure 11C,D). The conclusions were consistent between the validation and training datasets (Figure 11E–H). 

To investigate whether the association of the risk score with survival was related to age, site, or sex, univariate and multivariate Cox regression analysis were performed. Both univariate and multivariate analysis of the training dataset showed that risk score was significantly associated with survival, indicating that risk score can be used to independently predict the survival of ALS patients regardless of the value of other factors. In addition, age and site were also related to survival (Table 2). Univariate Cox regression analysis of the validation dataset showed that risk score was significant. Multivariate Cox regression analysis of the validation dataset showed that *p* < 0.1 for the risk score, indicating that it may be useful for independently predicting the survival of ALS patients regardless of the value of other factors (Table 3). Taking the results together, risk score may be an independent prognostic factor for ALS patients. 

## 4. Discussion

ALS is a fatal neurodegenerative disease that is characterized by the progressive degeneration of upper and lower motor neurons, and even neuronal death. The pathogenesis of ALS is a complex process involving multiple genes and pathways. The exact pathogenesis remains unclear. However, studies have shown that neuroinflammation is an important mediator of disease progression in patients with ALS, and it is mainly characterized by reactive microglia and astrocytes in the central nervous system [10]. Excess production of reactive oxygen species (ROS) is also an important pathological feature of ALS [25]. Mitochondrial dysfunction is a common feature of many neurodegenerative diseases, including ALS [25]. Disruption of mitochondrial structure, dynamics, bioenergy, and calcium buffering are considered to be directly involved in the pathogenesis of ALS [14]. In this study, we used systematic bioinformatics analysis to analyze the DEGs in the nerve tissue, lymphoid tissue, and whole blood. Our conclusion was consistent with previous conclusions. KEGG analysis revealed that the up-regulated genes in the nerve tissue of ALS patients were related to immunity and inflammation. We performed GSEA to validate the functions of the up-regulated genes in the nerve tissue. GSEA involving KEGG pathways showed that positive was mainly related to immunity, inflammation, and cell phagocytosis, and negative was mainly related to oxidative phosphorylation. GSEA involving GO BP terms revealed that positive was mainly related to immunity and inflammation, and negative was mainly related to energy metabolism and redox processes. The up-regulated genes co-existing in the nerve tissue, lymphoid tissue, and whole blood were related to immunity and inflammation, while the corresponding down-regulated genes were related to oxidation–reduction process and proteolysis. In conclusion, immune disorder, inflammation, mitochondrial dysfunction, and redox imbalance play important roles in the pathogenesis of ALS. 

**Figure 12:**
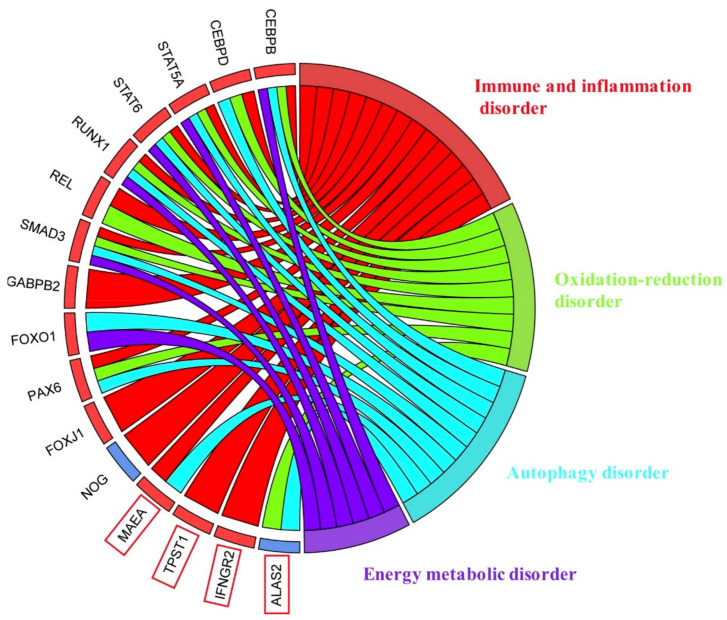
Summary of the relationship between 12 important transcription factors, 4 important survival-related markers and the potential pathogenic mechanism of ALS.

In this study, we found 12 transcription factors (STAT5A, STAT6, RUNX1, REL, SMAD3, CEBPB, CEBPD, GABPB2, FOXO1, PAX6, FOXJ1 and NOG) that are extremely important for the pathogenesis of ALS and four key prognostic markers (TPST1, IFNGR2, MAEA, ALAS2) (Figure 12). 

Neuroinflammation is one of the most typical features of ALS. All STAT family members (STAT1, STAT2, STAT3, STAT4, STAT5A, STAT5B, and STAT6) are related to autoimmune diseases [26]. Signal transducer and activator of transcription 5 (STAT5) is a transcription factor recruited by a variety of cytokines that plays an important role in maintaining a variety of physiological functions, including tissue growth, breast development, immune system activity, and lipid metabolism [27]. In innate immunity, STAT5A participates in the response of B cells to cytokines through the suppressor of cytokine signaling (SOCS) gene [28]. STAT5A is also necessary for Th2 cell differentiation and allergic airway inflammation [29]. STAT5A expression in CD4+ T cells is necessary for Th2 cell differentiation, and STAT5A participates in the development of CD4+CD25+ immunomodulatory T cells, which regulate the differentiation of T helper cells into Th2 cells [30]. JAK/STAT5 signaling plays a key role in regulating the intestinal response to infection and inflammation. Modulating internal STAT5 can protect the gastrointestinal tract from injury in gastrointestinal diseases [31]. The STAT5 regulatory mechanism in mast cells is important for the pathogenesis of atopic dermatitis [32]. The transcription factor STAT6 is crucial for activating cytokine gene expression and cytokine signaling in immune cells and target tissue cells [33]. STAT6 activation in microglia/macrophages has been observed in the ischemic areas of a stroke mouse model and stroke patients, and STAT6 activation in microglia/macrophages is crucial for neuroprotection [34]. IL-4 may partially promote M2 microglia/macrophage polarization through the JAK1/STAT6 pathway, therefore reducing neuroinflammation after intracerebral hemorrhage [35]. The absence of STAT6 signaling exacerbates inflammation, which is obviously the result of impaired generation of M2 anti-inflammatory macrophages [36]. Another polarization-specific epigenetic feature involving the IL-4/STAT6 signaling pathway decreases the macrophage response to inflammatory stimulation [37]. STAT6 regulates many pathological features of the pneumonia response in animal models, including airway eosinophilia, Th2 cell differentiation, and IgE production by B cells. IL-4 and IL-13, the cytokines upstream of STAT6, increased in human asthma, and clinical trials targeting the IL-4/IL-13/STAT6 pathway are ongoing [38]. STAT6 is activated by cytokines IL-4 and IL-13 and mediates the occurrence of allergic diseases, such as asthma, atopic dermatitis, and eosinophilic esophagitis [33]. In the development of allergic airway inflammation, STAT6 activation is very important for Th2 cell-mediated IgE production and the development of airway inflammation and hyperresponsiveness. Interference with STAT6 can effectively prevent the development of the main features of allergic airway diseases, such as allergen-induced airway inflammation and airway hyperreactivity [39,40]. JAK-STAT6 signaling can aggravate ovalbumin-induced asthma in mice, and STAT6-/- mice are highly resistant to allergic pulmonary inflammation [41,42]. STAT6 activation was detected in the inflamed colonic epithelium of patients with active inflammatory bowel diseases, and the activated STAT6 destroyed the tight junction integrity in the colonic epithelium. Compared to wild-type mice, STAT6-/- mice had a reduced colonic inflammatory response, reduced COX2 and intranuclear β-catenin concentration, and decreased IL17A and TNFα mRNA expression, but increased IL10 expression [43,44]. These findings suggest that STAT6 is a potential target for the treatment and prevention of inflammatory bowel diseases and enteritis. Taking these findings together, STAT5A and STAT6 play important roles in immune- and inflammation-related diseases. Our results showed that STAT5A and STAT6 were highly expressed in the nerve tissue of ALS patients, which may be closely related to the inflammatory process in the neural tissue of ALS patients and may play important roles in the pathogenesis of ALS. 

*REL* encodes the NF-κB subunit c-Rel. The NF-κB/REL transcription factor family plays a central role in the initiation and regression of the inflammatory response [45]. There are three important transcription factors (NF-κB, REL, and NF-κB1) involved in inflammation that may play important roles in the pathogenesis of ALS [46]. NF-κB is the main regulator of inflammation, and it is up-regulated in the spinal cord of ALS patients and SOD1-G93A mutant mice. Neuroinflammation caused by microglia activation is the key factor leading to the death of motor neurons, and prevention of NF-κB signaling in microglia prolongs the survival of ALS mice [47]. NF-κB signaling activation is also closely related to excessive aggregation of TDP-43, c9orf72, and FUS. The cytoplasmic accumulation of TDP-43 is a pathologic hallmark of ALS, and it is closely related to the expression of neuroinflammatory cytokines associated with NF-κB up-regulation [48]. Knockout of c9orf72 can activate the NF-κB pathway, suggesting the possibility that NF-κB participates in c9orf72-dependent immune responses [49]. FUS may act as a co-activator of p65 because it can enhance NF-κB responses to stimuli such as overexpression of TNFα, IL1β, and NF-κB-inducing kinase [50]. Taking these results together, NF-κB signal transduction plays an important role in the pathogenesis of ALS. Our findings indicated that NF-κB signaling was significantly activated in ALS patients, which further confirms the important role of inflammatory responses caused by NF-κB signaling in the pathogenesis of ALS. 

Runt-related transcription factor 1 (RUNX1) is a highly conserved transcription factor that regulates embryonic development, angiogenesis, hematopoiesis, immune response, and inflammatory response [51]. RUNX1 interacts with the IκB kinase complex in the cytoplasm or with NF-κB subunit P50 to participate in the regulation of the NF-κB signaling pathway, which plays a key role in lipopolysaccharide (LPS)-induced lung inflammation [51]. RUNX1 binds to P50 in macrophages and enhances TLR4-triggered inflammation and septic shock, suggesting that RUNX1 enhances NF-κB signaling [52]. Overexpression of RUNX1 promoted LPS-induced production of IL-1β and IL-6. Silencing of RUNX1 attenuated LPS-induced production of IL-1β and IL-6. A RUNX1 inhibitor also protected mice from LPS-induced endotoxic shock and greatly reduced the level of IL-6 [52]. These findings suggest that RUNX1 participates in immune and inflammatory responses by regulating NF-κB signaling. Moreover, NF-κB signaling has been confirmed to be closely related to the pathogenesis of ALS, which indicates the important role of RUNX1 in ALS. 

SMAD family member 3 (SMAD3) is a key mediator of the typical TGF-β signaling pathway and plays an important role in TGF-β1-mediated transcriptional regulation [53]. Phosphorylated SMAD3 translocation into the nucleus was found in sALS and fALS patients [54]. Double immunofluorescence staining of pSMAD3 and TDP-43 revealed that pSMAD3 and TDP-43 co-localize in round hyaline inclusions in sALS patients [54]. TGF-β1 mRNA, total SMAD3 protein level, and pSMAD3-positive nuclei increased in the skeletal muscle of symptomatic hSOD1-G93A mutant mice [55]. These findings suggest that activation of SMAD3 signaling may also play an important role in the pathogenesis of ALS. 

The transcription factor CCAAT enhancer-binding protein β (CEBPB) can be activated by a variety of inflammatory stimuli, including IL-17 and LPS, and it regulates many genes involved in inflammation [56]. It is a key driver of experimental autoimmune encephalomyelitis (EAE) autoimmune inflammation, and infiltration of lymphocytes and antigen-presenting cells into the central nervous system decreased in CEBPB-/- mice after EAE induction [56]. CEBPB was up-regulated in patients with Alzheimer’s disease, which increased the expression of pro-inflammatory genes in microglia, and the ubiquitin ligase COP1 inhibited neuroinflammation by silencing CEBPB in microglia [57]. It is noteworthy that CEBPB deletion inhibits the production of IL6 and prevents the pro-inflammatory phenotype [57,58]. Silencing CEBPB can inhibit the activation of the TNF-α signaling pathway and apoptosis [59,60]. CEBPB inactivation regulates the formation of macrophage foam cells in atherosclerosis by reducing inflammation, endoplasmic reticulum stress, and apoptosis and promoting autophagy [61]. In an Alzheimer’s disease model, blocking BDNF/TrkB neurotrophic signaling up-regulated inflammatory factors and activated the JAK2/STAT3 pathway, therefore up-regulating CEBPB [62]. Both CEBPB and CEBPD are key FcγR-mediated regulators of cytokine and chemokine production in macrophages [63]. CEBPB is highly expressed in M1-polarized macrophages, and CEBPD promotes M2 polarization of macrophages through IL-4/IL-13 [64]. CEBPD changes the Th17/Treg balance in an IL-10-dependent manner, and it plays a functional role in dendritic cells and central nervous system autoimmune diseases [65]. Moreover, CEBPD was highly expressed in the lymphocytes of ALS patients, so it can be used as an index to assess the progression of ALS [66]. Consistent with previous findings, our results showed that CEBPB and CEBPD were highly expressed in the nerve tissue of ALS patients, which may be closely related to the inflammatory process in the nerve tissue of these patients.

GA-binding protein (GABP) is a widely expressed ETS family transcription factor that is composed of two subunits: the DNA-binding GABP α subunit and the transactivation GABP β subunit [67]. GABP regulates many genes responsible for regulating mitochondrial energy metabolism, including cytochrome c oxidase, ATP synthase, and a subunit of mitochondrial transcription factor 1 [68]. Forkhead box transcription factor O1 (FOXO1) is an important transcription factor involved in the activation of neuronal autophagy [69]. Mitogen-activated protein kinase kinase kinase kinase 4 (MAP4K4) inhibition has been shown to reduce motoneuron death in ALS partly by activating FOXO1-mediated autophagy and therefore reducing the accumulation of protein aggregates [70]. FOXJ1 is one of the members of the Forkhead/winged-helix (FOX) family of transcription factors. It is necessary for the postnatal differentiation of ependymal cells and subventricular astrocyte subsets. Its expression increases gradually after brain injury, which may be involved in the pathophysiological processes after traumatic brain injury [71]. Paired box 6 (PAX6) is a multifunctional transcription factor that regulates the expression of genes involved in cell proliferation, differentiation, inflammation, oxidative stress, and neuropathy [72]. It has been shown to regulate brain immunity by activating microglia [73]. It may directly or indirectly bind to the promoters of genes necessary for brain immune surveillance and energy metabolism that exhibit altered expression during aging [74]. The roles of GABPB2, FOXO1, PAX6, and FOXJ1 in the pathogenesis of ALS need to be further studied. 

Noggin (NOG), which is a member of the TGF-β superfamily, is a bone morphogenetic protein (BMP) antagonist that is related to nerve recovery and nerve regeneration [75]. NOG treatment can promote the transformation of microglia from the M1 phenotype to the M2 phenotype in ischemic stroke (that is, M1 markers of activated microglia decrease and M2 markers increase) [76]. Treatment with NOG-modified bone marrow stromal cells inhibited ischemia-induced apoptosis and inflammatory responses in a rat model of brain ischemia [77]. There was a high level of IgG autoantibodies against NOG in patients with ankylosing spondylitis, which may be an important pathogenic mechanism of ankylosing spondylitis [78]. In human hepatoma cells, IL-6 up-regulated hepcidin, which was inhibited by NOG [79]. In addition, NOG treatment greatly reduced the mRNA and protein expression of various inflammatory molecules in diabetic mice (such as VCAM-1, ICAM-1, CTGF, and BMP-4) [80]. NOG was hypermethylated in Alzheimer’s disease tissue, and it was significantly down-regulated in Alzheimer’s disease tissue compared to the control group [81]. Taking these results together, low NOG expression is closely related to the occurrence of inflammation. Our results showed that NOG was very weakly expressed in the nerve tissue of ALS patients, which may be closely related to neuroinflammation, and NOG may be an important regulator in the pathogenesis of ALS. 

Our results revealed that high TPST1, IFNGR2, and MAEA expression and low ALAS2 expression were closely related to short survival in ALS patients. Tyrosylprotein sulfotransferase 1 (TPST1) transfers sulfate moieties to proteins that are then secreted, using 3′phosphoadenosine-5′phosphosulfate (PAPS) as the donor [82], in the trans-Golgi network. Several tyrosine sulfation targets (9 of 62 identified targets) are cell adhesion molecules and chemokine receptors, which are core participants in leukocyte transport [83]. Neutrophils express TPST1, which is one of the five sulfotransferases related to synthesis of the selectin ligand [84]. TPST1 plays a role in LPS-induced production of pro-inflammatory cytokines in macrophages, and TPST1 down-regulation inhibits LPS-induced IL-6 production [85]. TPST1 is up-regulated in bone marrow-derived monocytes from patients with rheumatoid arthritis, which is related to the immune response [83]. Taking these results together, TPST1 may play an important role in the occurrence and development of inflammation. TPST1 up-regulation is related to high pathological staging and low survival rate in patients with urothelial bladder cancer [86] and a decreased level of tissue factor pathway inhibitor (TFPI) [87]. However, the role of TPST1 up-regulation in the whole blood of ALS patients still needs to be further investigated. 

The Th1 cytokine interferon (IFN)-γ is a pleiotropic, pro-inflammatory cytokine that functions through two IFN-γ receptors: IFNGR1 and IFNGR2 [88]. IFN-γ plays a role in the induction/proliferation of Th1 cells through these receptors, which may inhibit the Th2 response; this may be the basis of atopic asthma [89]. IFNGR2 has been identified as a potential causal gene for two immune-related diseases: rheumatoid arthritis and psoriasis [90,91]. IFNGR2Q64R polymorphism is associated with male sex and paranoid schizophrenia, and chronic neuroinflammation may be a factor that induces the development of paranoid schizophrenia in men [92]. At 7 and 14 days after mesenteric tumor inoculation, *IFNGR2* expression increased in mesenteric white adipose tissue and played a role in cancer cachexia-related systemic inflammation [93]. In a BALB/c mouse model of dextran sodium sulfate-induced colitis, *IFNGR2* was significantly up-regulated [94]. Patients with inclusion body myositis had segmental IFNGR2 up-regulation in myofibroblast membranes, which was positively correlated with the number of adjacent CD8+ T cells [95]. In Alzheimer’s disease, IFN-γ couples with choroidal–cerebral neuroimmune interactions, and a variety of IFN signaling-related genes, including *IFNGR2*, are up-regulated, suggesting that *IFNGR2* may be involved in the pathogenesis [96]. Our results showed that IFNGR2 was highly expressed in the whole blood of ALS patients, and it was related to poor prognosis, but its exact mechanism still needs to be further investigated.

Macrophage erythroblast attacher (MAEA) encodes a protein that plays a role in erythroblast enucleation and the normal differentiation of erythroid cells and macrophages. It regulates the development of mature macrophages by mediating attachment to erythroblasts and promotes adult bone marrow erythropoiesis by regulating the maintenance of macrophages [97,98]. MAEA is also an agonist of cannabinoid receptor 1 (CB1), which mediates macrophage phagocytosis [99]. MAEA is a membrane-associated E3 ubiquitin ligase subunit that is essential for the maintenance of hematopoietic stem cells and lymphatic potential; deletion of MAEA increases the expression of surface cytokine receptors in hematopoietic stem cells and impairs autophagy flux [100]. MAEA overexpression in primary mouse liver cells attenuates hepatic gluconeogenesis and reduces the risk of insulin resistance, which may improve type 2 diabetes mellitus [101,102]. MAEA is associated with low bone mineral density in postmenopausal women [103]. In summary, the functions of MAEA are very complex. The role of MAEA in ALS is still unclear and further investigation is needed. 

5-Aminolevulinate synthase 2 (ALAS2) is a rate-controlling enzyme of erythroid heme synthesis [104]. ALAS2 knockout can affect erythrocyte differentiation by down-regulating the mitochondrial autophagy receptor BNIP3L [105]. ALAS2 mutation is usually associated with sideroblastic anemia, iron overload, and X-linked macrocytic erythropoietic anemia in women [106,107]. ALAS2 deficiency itself does not interfere with the development of final erythroid cells, but it leads to the accumulation and peroxidation of a large amount of iron in erythrocytes [108]. ALAS2 mRNA is weakly expressed in bone marrow erythrocytes of patients with congenital sideroblastic anemia [109]. In human erythroid leukemia cells, ALAS2 knockdown inhibits erythroid differentiation and alters iron metabolism [110]. Up-regulation of α-synuclein (SNCA) can induce autosomal dominant Parkinson’s disease, and SNCA and ALAS2 are co-expressed, suggesting that ALAS2 may play an important role in the pathogenesis of Parkinson’s disease [111]. In conclusion, low ALAS2 expression is related to erythroid differentiation and iron metabolism. Our results showed that ALAS2 was weakly expressed in the whole blood of ALS patients, which affects the oxygen carrying capacity of red blood cells and causes relative hypoxia. Severe tissue hypoxia can not only produce a large amount of reactive oxygen species and cause redox imbalance and inflammation, but it also inhibits energy production, further aggravating tissue damage, which may play an important role in the pathogenesis of ALS. However, its exact role in the pathogenesis of ALS remains to be further investigated.

## 5. Conclusions

In this study, we analyzed the function of the DEGs in nerve tissue, lymphoid tissue, and whole blood of ALS patients and screened for biomarkers related to the survival of ALS patients. We found that the up-regulated genes in ALS were mainly involved in the regulation of immune and inflammation-related processes, and the down-regulated genes in ALS were mainly involved in the regulation of energy metabolism and redox processes. We also found 11 up-regulated transcription factors (CEBPB, CEBPD, STAT5A, STAT6, RUNX1, REL, SMAD3, GABPB2, FOXO1, PAX6, and FOXJ1) and one down-regulated transcription factor (NOG) in the nerve tissue of ALS patients. In addition, MAEA, TPST1, IFNGR2, and ALAS2 were greatly related to the survival of ALS patients. High expression of MAEA, TPST1, and IFNGR2 leads to short survival of ALS patients, while high expression of ALAS2 results in long survival of ALS patients. Taken together, our findings provide evidence for the determination of the pathogenic mechanism of ALS, potential targets for new drug development, and new markers for predicting the prognosis of ALS.

## Figures and Tables

**Table 1:** Important regulatory factors in pathogenesis of ALS.

Gene	Degree	Outdegree	*p*-Value	FDR	Fold Change
CEBPD	28	25	1.02 × 10^−6^	1.05 × 10^−4^	3.9261
STAT6	41	38	1.37 × 10^−5^	0.001371759	3.7290
RUNX1	63	58	3.24 × 10^−14^	3.57 × 10^−12^	3.3537
GABPB2	51	50	7.13 × 10^−5^	0.006990821	3.1583
CEBPB	70	67	0	0	2.9424
FOXO1	40	39	5.90 × 10^−8^	6.20 × 10^−6^	2.6586
PAX6	20	14	7.42 × 10^−6^	7.49 × 10^−4^	2.5465
REL	23	22	4.63 × 10^−5^	0.00458507	2.4250
STAT5A	87	85	0	0	2.2199
FOXJ1	44	44	6.03 × 10^−9^	6.39 × 10^−7^	2.1753
SMAD3	26	25	6.15 × 10^−6^	6.28 × 10^−4^	2.0401
NOG	53	53	1.30 × 10^−7^	2.34 × 10^−6^	0.4031

Legend: FDR, false discovery rate.

**Table 2:** Univariate Cox regression analysis and multivariate Cox regression analysis on the training dataset.

	Univariate Cox Regression Analysis				Multivariate Cox Regression Analysis			
	Coef	HR	(95% CI.HR)	*p*-Value	Coef	HR	(95% CI.HR)	*p*-Value
age_status	0.74	2.1	(1.6–2.7)	1.1 × 10^−9^	0.677958	1.969852	(1.52–2.54)	1.92 × 10^−7^
site_onset	−0.49	0.61	(0.48–0.79)	0.00016	−0.47589	0.621334	(0.48–0.80)	2.48 × 10^−4^
risk_status	0.51	1.7	(1.3–2.1)	6.5 × 10^−5^	0.440701	1.553797	(1.2–2.0)	6.82 × 10^−4^
sex	0.028	1	(0.84–1.3)	0.78	−0.05221	0.949129	(0.64–1.15)	0.601904

Legend: Coef, coefficient; HR, hazard ratio; CI, confidence interval.

**Table 3:** Univariate Cox regression analysis and multivariate Cox regression analysis on the validation dataset.

	Univariate Cox Regression Analysis				Multivariate Cox Regression Analysis			
	Coef	HR	(95% CI.HR)	*p*-Value	Coef	HR	(95% CI.HR)	*p*-Value
age_status	0.41	1.5	(0.95–2.4)	0.082	0.40534076	1.49	(0.90–2.48)	0.121
site_onset	0.0029	1	(0.62–1.6)	0.99	0.02816389	1.02	(0.61–1.72)	0.562
risk_status	0.48	1.6	(1–2.6)	0.044	0.42652788	1.53	(0.94–2.48)	0.084
sex	0.079	1.1	(0.72–1.6)	0.71	0.13604963	1.14	(0.75–1.74)	0.526

Legend: Coef, coefficient; HR, hazard ratio; CI, confidence interval.

## Data Availability

Data are contained within the article.

## Supplementary Materials

The following are available online at https://www.mdpi.com/article/10.3390/antiox11020303/s1, Figure S1: Heatmap of the expression of 79 characteristic genes in the ALS and control samples in the training dataset, Figure S2: Heatmap of the expression of 79 characteristic genes in the ALS and control samples in the validation dataset, Table S1: mRNA microarray and sequencing in patients with ALS, Table S2: Characteristic genes (79), Table S3: Evaluate the prediction results of the model in sample classification, Table S4: Survival-related markers screened by univariate Cox proportional hazards regression, Table S5: Random survival forests algorithm screen the most relevant marker for survival.
