# Supplementary material for: Identification of Regulatory Factors and Prognostic Markers in Amyotrophic Lateral Sclerosis
Source: Antioxidants (Basel). 2022 Feb 1;11(2):303. doi: 10.3390/antiox11020303 (PMC8868268; doi:10.3390/antiox11020303)
Supplement: Supplementary file 1 [file antioxidants-11-00303-s001.zip › antioxidants-1551421-supplementary/Supplementary Table S1-revised.pdf]

Supplementary Table S1. mRNA microarray and sequencing in patients with ALS.

GEO ID	Sample type	Platform	Sample Size	Molecular type
GSE26927	Snap-frozen brain blocks	Illumina humanRef-8 v2.0	20	mRNA
GSE76220	Motor neuron of lumbar spinal cord	Sequencing	80	mRNA
GSE67196	Frontal Cortex<br>Cerebullum	Sequencing	19<br>18	mRNA
E-MTAB-2325	Motor cortext	Agilent 4*44k	41	mRNA
GSE112681	Whole blood	Illumina HT	1117	mRNA
E-GEOD-28253	Lymphocytes	Agilent 4*44k	22	mRNA

Legend: ALS, Amyotrophic Lateral Sclerosis; GEO, Gene Expression Omnibus; ID, Identity Document.
