# Supplementary material for: Identification of Regulatory Factors and Prognostic Markers in Amyotrophic Lateral Sclerosis
Source: Antioxidants (Basel). 2022 Feb 1;11(2):303. doi: 10.3390/antiox11020303 (PMC8868268; doi:10.3390/antiox11020303)
Supplement: Supplementary file 1 [file antioxidants-11-00303-s001.zip › antioxidants-1551421-supplementary/Supplementary Table S3-revised.pdf]

Supplementary Table S3. Evaluate the prediction results of the model in sample classification.

Classifier	Training			Validation			All		
	Accuracy	Precision	Recall	Accuracy	Precision	Recall	Accuracy	Precision	Recall
**SVM**	0.996	0.993	0.997	0.94	0.916	0.926	0.982	0.974	0.979
**Randomforest**	1	1	1	1	1	1	1	1	1
**Adaboost**	1	1	1	0.931	0.897	0.926	0.983	0.974	0.981

Legend: SVM, support vector machines.
