# Supplementary material for: Identification of Regulatory Factors and Prognostic Markers in Amyotrophic Lateral Sclerosis
Source: Antioxidants (Basel). 2022 Feb 1;11(2):303. doi: 10.3390/antiox11020303 (PMC8868268; doi:10.3390/antiox11020303)
Supplement: Supplementary file 1 [file antioxidants-11-00303-s001.zip › antioxidants-1551421-supplementary/Supplementary Table S4-revised.pdf]

Supplementary Table S4. Survival related markers screened by univariate Cox proportional hazards regression.

	Coef	HR(95 CI for HR)	p.value	FDR
ABCA1	0.25	1.3 (1-1.6)	0.02	0.121538
TMEM71	0.26	1.3 (1-1.6)	0.028	0.13825
VNN2	0.33	1.4 (1.1-1.8)	0.0082	0.080975
RGS2	0.18	1.2 (1-1.4)	0.027	0.13825
SRPK1	0.32	1.4 (1.1-1.8)	0.018	0.121538
USP32	0.27	1.3 (1-1.7)	0.025	0.13825
March1	0.36	1.4 (1.1-1.8)	0.0038	0.077683
TPST1	0.33	1.4 (1.1-1.8)	0.0058	0.077683
SLC31A2	0.35	1.4 (1.1-1.9)	0.012	0.0948
TLE4	0.33	1.4 (1.1-1.8)	0.0069	0.077871
FOS	0.28	1.3 (1.1-1.7)	0.012	0.0948
FGL2	0.28	1.3 (1-1.8)	0.049	0.215056
MAEA	0.39	1.5 (1.1-2)	0.019	0.121538
GLIPR1	0.45	1.6 (1.1-2.1)	0.0059	0.077683
IFNGR2	0.59	1.8 (1.3-2.5)	0.00044	0.03476
CASP4	0.37	1.4 (1-2)	0.03	0.139412
D2HGDH	-0.42	0.66 (0.49-0.87)	0.004	0.077683
ALAS2	-0.18	0.83 (0.74-0.94)	0.0033	0.077683

Legend: Coef, coefficient; HR, hazard ratio; CI, confidence interval; FDR, False Discovery Rate.
