# Supplementary material for: Identification of Regulatory Factors and Prognostic Markers in Amyotrophic Lateral Sclerosis
Source: Antioxidants (Basel). 2022 Feb 1;11(2):303. doi: 10.3390/antiox11020303 (PMC8868268; doi:10.3390/antiox11020303)
Supplement: Supplementary file 1 [file antioxidants-11-00303-s001.zip › antioxidants-1551421-supplementary/Supplementary Table S5.pdf]

Supplementary Table S5. Random survival forests algorithm screen the most relevant marker for survival.

Name	Importance	Absolute Value	Relative Importance
TPST1	1.09E-02	1.09E-02	1.0
ALAS2	8.16E-03	8.16E-03	0.7
IFNGR2	6.97E-03	6.97E-03	0.6
MAEA	6.68E-03	6.68E-03	0.6
TMEM71	3.59E-03	3.59E-03	0.3
VNN2	2.90E-03	2.90E-03	0.3
FGL2	-2.39E-03	2.39E-03	0.2
RGS2	-2.25E-03	2.25E-03	0.2
USP32	2.04E-03	2.04E-03	0.2
FOS	1.76E-03	1.76E-03	0.2
GLIPR1	1.45E-03	1.45E-03	0.1
D2HGDH	-1.00E-03	1.00E-03	0.1
ABCA1	4.55E-04	4.55E-04	0.0
TLE4	4.15E-04	4.15E-04	0.0
CASP4	3.08E-04	3.08E-04	0.0
SRPK1	-2.80E-04	2.80E-04	0.0
SLC31A2	5.99E-05	5.99E-05	0.0
March1	5.15E-05	5.15E-05	0.0
