# Supplementary figures and images for: Identification of Regulatory Factors and Prognostic Markers in Amyotrophic Lateral Sclerosis
Source: Antioxidants (Basel). 2022 Feb 1;11(2):303. doi: 10.3390/antiox11020303 (PMC8868268; doi:10.3390/antiox11020303)

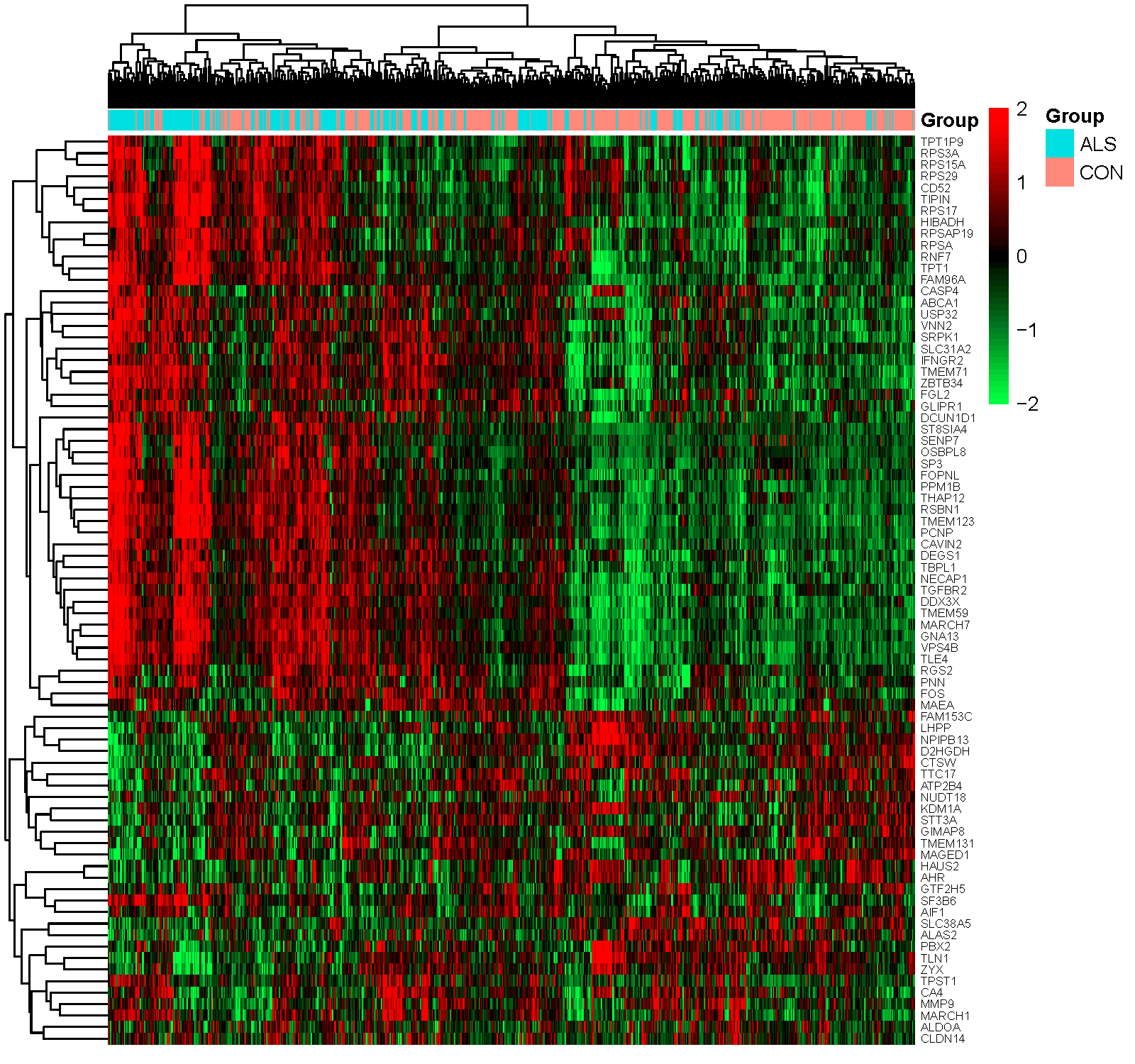

Supplement: Supplementary file 1 [file antioxidants-11-00303-s001.zip › antioxidants-1551421-supplementary/Supplementary Figure S1.TIFF]

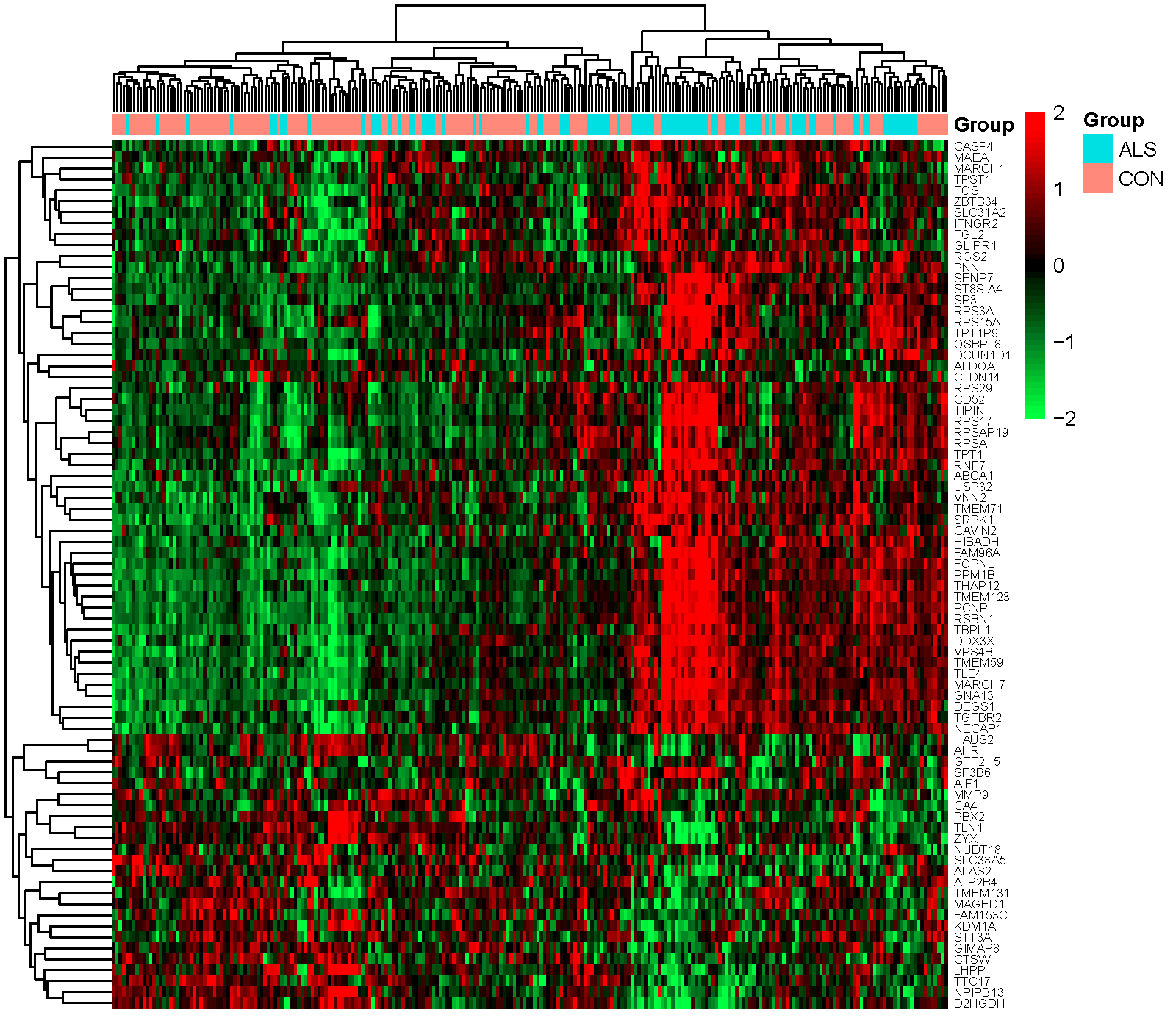

Supplement: Supplementary file 1 [file antioxidants-11-00303-s001.zip › antioxidants-1551421-supplementary/Supplementary Figure S2.TIFF]
